# Cancer genetics program: Follow‐up on clinical genetics and genomic medicine in Qatar

**DOI:** 10.1002/mgg3.534

**Published:** 2018-12-16

**Authors:** Salha Bujassoum Al‐Bader, Reem Alsulaiman, Hekmet Bugrein, Tawfeg Ben Omran, Fatemeh Abbaszadeh, Nawal Bakheet, Sitti Apsa Kusasi, Nema Abdou, Benjamin D. Solomon, Hafedh Ghazouani

**Affiliations:** ^1^ Department of Medical Oncology, National Center of Cancer Care and Research Hamad Medical Corporation Doha Qatar; ^2^ Section of Clinical and Metabolic Genetics, Department of Pediatrics Hamad Medical Corporation Doha Qatar; ^3^ Weill Cornell Medical College Doha Qatar; ^4^ Sidra Medicine Doha Qatar; ^5^ Diagnostic Molecular Laboratory Hamad Medical Corporation Doha Qatar; ^6^ GeneDx Gaithersburg Maryland

## Abstract

This article presents an overview of the cancer genetics program in Qatar. In addition to summarizing clinical, research, educational, and other aspects, data related to testing outcomes (over the course of approximately 5.5 years) are presented.

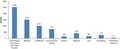

## 1 INTRODUCTION

Cancer is a genetic disease which causes alterations in the cell's makeup and replication system leading to uncontrolled growth. Although most cases of cancers are thought to be sporadic and multifactorial in etiology, about 5%–10% of cancers is hereditary and is due to germline mutations in cancer predisposition genes. These hereditary cancers are often (but not always) observed in families with positive family history of the relevant cancer types, and frequently involve features such as young age at diagnosis in addition to specific histopathological features. With the advances in field of genomics and precision medicine, it is now feasible to identify patients at risk of cancer due to hereditary predisposition and offer appropriate genetic counseling, risk‐reducing strategies, and targeted therapies to decrease their risk of developing cancer and/or improve their treatment management based on the identified cancer syndrome. The recognition of several cancer syndromes and the establishment of management guidelines have led to a model, at least in some medical centers, as standardly incorporating cancer genetics as part of the practice of the medical oncology subspecialty (Burke, Daly, et al., 1997; Burke, Petersen, et al., 1997) This article details an example of such a practice in the State of Qatar, including outcomes to date.

The National Center for Cancer Care and Research (NCCCR) is the premier and only cancer hospital in the State of Qatar. It is part of Hamad Medical Corporation (HMC), which is the main governmental hospital, and which is dedicated to providing services to cancer patients who require ongoing treatments such as chemotherapy, targeted and hormonal treatment, and immunotherapy in addition to radiotherapy. The NCCCR also treats benign and malignant hematologic disorders and offers bone marrow transplants in addition to palliative care and pain management. Surgical oncology services are delivered in its partner hospital, Hamad General Hospital.

## 2 CANCER GENETICS PROGRAM AT NCCCR, QATAR

Due to the incidence of early cancer onset and the identification of several patients with hereditary breast and ovarian cancer syndromes, the cancer genetics program have been incorporated into routine cancer services starting March 2013 at the NCCCR under the direction of Dr. Salha Bujassoum Al‐Bader, a senior medical oncologist trained in cancer genetics and assisted by a full‐time board‐certified cancer genetics counselor Dr. Reem Alsulaiman.

The program was later recognized at the country's national cancer framework as one of the dedicated services in cancer care with the aim of reducing the incidence of cancer in high‐risk populations. This program is described as an example of an integrated multidisciplinary and well‐established service targeted toward achieving excellence in cancer care through offering prevention and personalized medicine; these goals align with Qatar's 2022 vision of achieving excellence in cancer care (Qatar’s National Cancer Framework, 2017–2020).

In addition to the management of patients who have already been diagnosed with cancer, this is the first regional program designed to manage unaffected, high‐risk patients, and their families—that is, individuals who are at increased risk for cancer due to medical and/or family histories. This program offers genetic risk assessment, genetic counseling, and the use of preventative strategies to attempt to reduce cancer risks.

From 2013 until 2015, the cancer genetics program primarily focused on patients with potential risk for hereditary breast and ovarian cancers. In 2016, the program expanded to encompass other patients at elevated risk of other cancers that can be of hereditary etiology, including but not limited to gastrointestinal, endocrine, gynecological, dermatological, urological, hematological, and other types of rare cancers, and cancer syndromes.

## 3 SERVICES

The program is supported by a multidisciplinary team of experts including medical oncologists, board‐certified cancer genetic counselors, collaborators from pediatric medical genetics, the molecular genetics laboratory at HMC, and collaborating genetics laboratories in other countries. Other experts who support the program include genetic counselor assistants (GCAs), nurses, psychologists, and dedicated experts in related specialties such as surgery, plastic surgery, gynecology, gastroenterology, endocrinology, urology, dermatology, and other specialties.

The program currently holds three weekly genetic counseling clinics, evaluating on average 6–12 patients per clinic, and two weekly clinics for surveillance is the latter of which is run by medical oncologists and which sees on average 12 patients per clinic. A visit to the cancer genetics clinic usually includes time with both a dedicated medical oncologist and a genetic counselor, both of whom have specific expertise in both common and rare inherited cancer syndromes. By working closely with referring physicians, the team designs individualized programs to monitor patients for the earliest signs of cancer as well as to institute preventative measures where possible.

Comprehensive services provided by the program include genetic counseling, risk assessment, genetic testing and, importantly risk‐reducing strategies including surveillance, chemoprevention, and risk‐reducing surgeries. The program also helps guide the patient's overall management in related ways. For example, genetic testing results can help determine eligibility for targeted therapies, which can benefit outcomes and reduce mortality in some situations. In addition, families with known familial mutations are offered reproductive options such as preimplantation genetic diagnosis (PGD) to help reduce the chances of passing down disease‐related genetic changes. This service is now available through the Assisted Conception Unit (ACU) at the Women's Hospital‐HMC, which collaboratively works with families wishing to pursue PGD.

Beyond providing direct care, the program establishes clinical practice guidelines and provides public education related to several hereditary cancers for clinicians and healthcare workers. The program has also participated in a number of educational meetings and research projects related to diverse aspects of cancer.

## 4 PROGRAM UNIQUENESS

The cancer genetics program in Qatar is the first of its kind in the region and serves a heterogeneous high‐risk population from different backgrounds and ethnicities. The Qatari population specifically has a unique genetic profile and patterns in disease presentation, and incidence, including familial clusters in addition to unique cultural aspects and characterized by a high consanguinity rate, large family size, and high prevalence of certain genetic disorders mainly due to founder effect. As with all modern societies, religious, cultural, and ideological beliefs play a central role in the practice of medicine and clinical genetics. However, due to small size of the Qatari population, high rate of consanguinity and the high rate of stigma toward cancer and genetic diseases, the program has been designed with extra care to maintain the confidentiality of patients and their families through heightening the privacy of medical records and medical registries and to provide culturally oriented counseling approaches while maintaining standardized and rigorous medical program competencies.

## 5 IMPACT OF THE PROGRAM ON PATIENTS AND OTHER STAKEHOLDERS

The services offered at the cancer genetic clinic impacts patients and their families’ medical care by identifying and offering targeted therapies as well as risk‐reducing strategies such as increased surveillance or prophylactic surgeries to reduce mortality and morbidity. Family relatives of the patient with hereditary cancer syndrome can benefit through receiving targeted genetic testing in addition to allowing reproductive options.

The cancer genetics program also impacts other stakeholders such as healthcare providers. When the cancer genetics program was first established, the program collated and distributed referral criteria to all departments of HMC as well as other primary health centers in the geographic region. As part of this education campaign, multiple didactic lectures have been given to different sectors of the healthcare system in order to raise awareness about the diagnosis and management of hereditary cancer

The establishment of the cancer genetics program has also yielded a successful and mutually beneficial collaboration with HMC molecular laboratory to establish implement one of the most frequently indicated genetic tests (germline molecular testing for the *BRCA1* and *BRCA2*). Performing this test locally is more convenient and cost‐effective than sending samples abroad for genetic testing. In addition, the establishment of the local program has enriched relationships with well‐known and advanced molecular laboratories abroad, to which the program often sends samples for additional panel‐based genetic testing, or, in some cases, whole exome sequencing (WES).

## 6 REFERRALS AND PATHWAY

At the cancer genetics program, referral criteria were established and distributed to different departments of HMC and to the primary health centers to help local providers identify and refer high‐risk patients early for further assessment and evaluation. Eligible patients include those affected with cancer below the age of 50 and those who might be older than 50 years of age but with cancers of specific histopathologies such as “triple‐negative” breast cancer, high‐grade serous ovarian cancer, medullary thyroid cancer, and mismatch‐repair deficient colon cancers, male breast cancer, polyposis, and the presence of multiple primary cancers. Patient who are also eligible to be referred to the program include those with extensive family histories of early‐onset cancers such as breast, ovarian, colon, prostate, pancreatic, and other cancers in addition to patients of high‐risk ethnicity.

From the program's experience, patients are often referred to the cancer genetics program from different departments at Hamad HMC, primary health centers, but also from other private hospitals and even from neighboring countries. Common referrals include individuals with known familial mutations, personal history of young‐onset cancers such as breast, ovarian, colon, endocrine cancers, or personal history of polyposis and individuals with family history of different malignancies.

**Figure 1:**
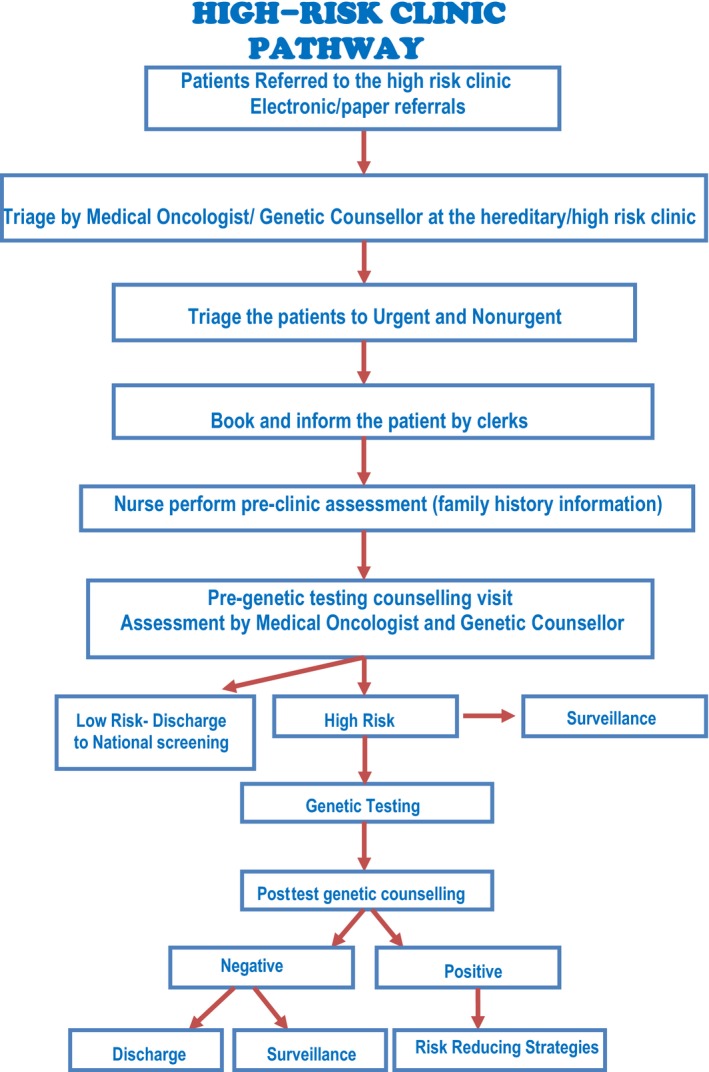
High‐risk clinic algorithm

All referrals are triaged by medical oncologists and genetic counselors; patients are stratified into urgent or non‐urgent by assessing patient's personal and family histories using information found in medical records and information provided by the referring clinicians. After patients this stratification, cancer program nurses acting as GCAs obtain more information directly from the patients, especially related to family history, which helps the medical oncologist and genetic counselors in their clinic assessments. Specifically designed family history and consent forms for pursuing and disclosing genetic testing results as well as educational pamphlets are used. After clinic evaluations, if patients are classified as high‐risk through international scoring systems and testing criteria, they are offered genetic testing and/or surveillance based on the overall assessment. If patients are classified as low‐risk, they are discharged to standard national screening. The pathway of the cancer genetics program is illustrated in Figure 1.

## 7 CLINIC VISITS AND HIGH‐RISK MDT

A visit to the cancer genetics clinic usually includes several clinical assessments by both the medical oncologist and the genetic counselor, both of whom have expertise in diverse forms of inherited cancer syndromes. GCAs with nursing backgrounds play a vital role in collecting family history information and in preparing patient's for the clinic visits.

Patients are typically first seen for a pre‐test genetic counseling visit and are assessed by both medical oncologists and genetic counselors (including through use of standard scoring/assessment tools) for both the risk for hereditary cancer as well as their risk of developing certain cancers. If patients are eligible for genetic testing, then they are comprehensively counseled by the genetic counselors on the implications of genetic testing on their and their families’ future risk of cancers, on possible outcomes of genetic testing, and management options in the case of a positive result.

Patients return for a second post‐test genetic counseling, visit, where genetic counselors explain and discuss the genetic testing results and their implications. If patients are found to be negative on genetic testing but with high lifetime risk of developing certain cancers due to family history, than an individualized surveillance program is proposed to the patient and the patients are then followed in the same clinic. In some cases, patients are not eligible for genetic testing but are at high risk of cancer due to familial risk, and then these individuals are placed under surveillance.

If testing results are positive, then the patients are often seen in a combined session by the genetic counselor and the medical oncologist for further discussion on risk‐reducing strategies including (as relevant to the specific situation) prophylactic surgery, chemoprevention, and surveillance. Patients are often referred to additional specialists for their surveillance and are also followed regularly by the cancer genetics program.

If patients are found to be positive by genetic testing and are affected with cancer, then the primary oncologist/surgeon/medical physicians are informed in order to plan proper high‐risk surveillance. Patients, where treatment management can be altered through genetic testing, are usually seen as urgent basis. Examples of these patients include patients with ovarian or breast cancers and *BRCA* pathogenic variants, who may be eligible to receive targeted therapies such as PARP inhibitors, or patients with locally advanced breast cancer on neoadjuvant chemotherapy, whose surgical decisions may be further influenced by testing results.

Family members of patients with positive findings are invited through the index case and are counseled and offered familial genetic testing by the genetic counselors. If positive, these patients are counseled appropriately regarding management options. In addition, families with pathogenic or likely pathogenic variants (together abbreviated as PV) undergo genetic counseling regarding reproductive options such as PGD and referred to the IVF/PGD department at HMC.

This collaborative approach allows medical oncologists and genetic counselors to work together to help provide patients with the most accurate and individualized risk assessment and risk‐reducing strategies based on genetic testing, personal and/or family histories in addition to contributing in guiding patient's treatment management.

### 7.1 High‐risk multidisciplinary team meeting (MDT)

The cancer genetic program have established a high‐risk MDT where all patients with positive findings are discussed by a team of experts including oncologists, geneticists and genetic counselors, surgeons, gynecologists, nurses, and psychologists in addition to other healthcare providers who are invited when needed to discuss the optimal risk‐reducing strategies for each patient. All risk‐reducing strategies including prophylactic surgeries, chemoprevention, and surveillance facilities are available locally at HMC.

## 8 GENETIC TESTING

In 2015, Hamad Medical Corporation molecular laboratory received College of American Pathologists (CAP) accreditation to perform *BRCA* testing in house, which made it easier for resident patients to get tested without cost.

However, for other single gene testing, panel genetic testing, or in some cases WES, samples are often sent abroad to collaborating laboratories.

The HMC molecular laboratory is now working to validate panel testing for other cancer genes and panels for multiple hereditary cancer syndromes.

## 9 SUMMARY OF CLINIC DATA FROM 2013 TO 2018

**Figure 2:**
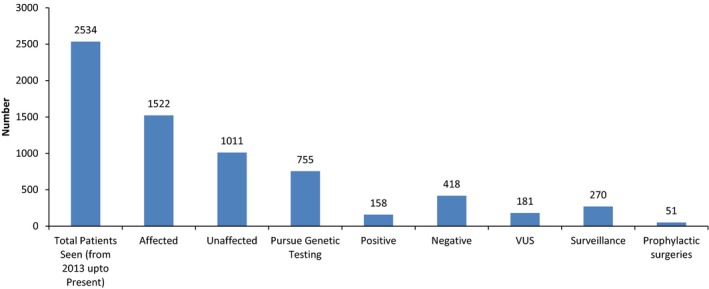
Summary of clinic data from March 2013 to September 2018

**Figure 3:**
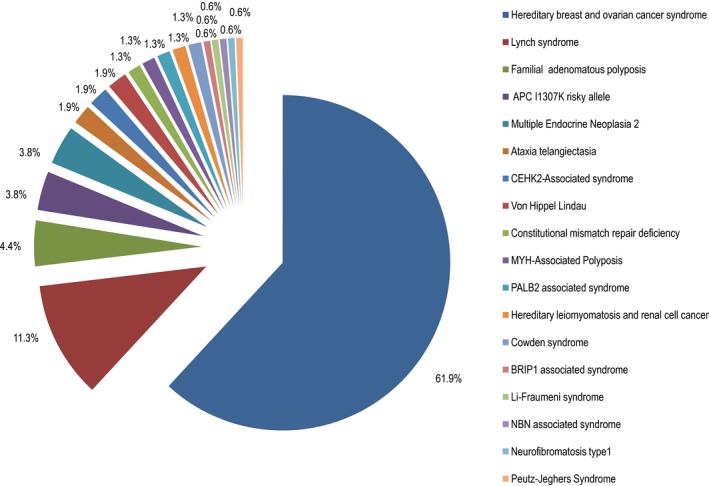
Hereditary cancer syndromes identified at the hereditary cancer program March 2013–September 2018

**Figure 4:**
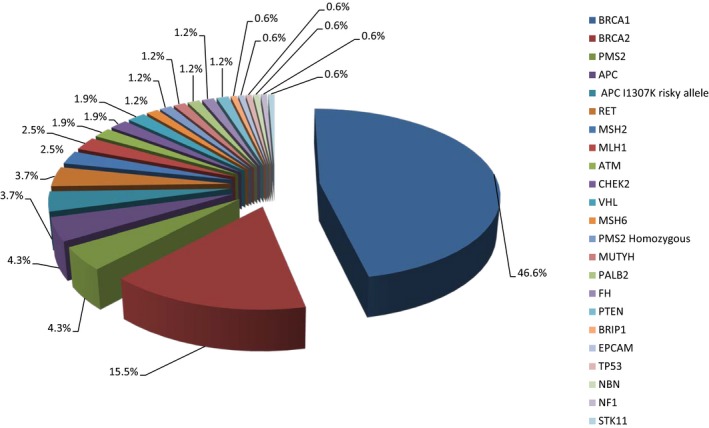
Hereditary cancer syndromes (divided by genes) identified at the hereditary cancer program March 2013–September 2018

Since the establishment of the program, a centralized cancer genetics registry was established where all patients are registered in the database and are stratified by families, starting by the index case. From our registry, we can illustrate a summary of the number of patients seen since the establishment of the program and the hereditary cancer syndromes identified at our program as can be seen in Figures 2, 3, 4.

From March 2013 to September 2018, a total of 2,534 of patients were assessed at the clinic (Figure 2). Of these, 1,522 (60%) were affected with or without family history, while 1,011 (40%) were unaffected but had a clinically significant family history of cancer. Following genetic counseling, 755 individuals (30%) pursued genetic testing. Of these 755, 158 (21%) were positive (had PVs identified), 418 (55%) were negative, and 181 (24%) had variants of unknown significance (VUS). Following assessment and testing, a total of 270 were placed under surveillance for breast, ovarian, colon, or other types of cancers due to genetic results or family history. A total of 51 patients underwent risk‐reducing prophylactic surgery. These included 13 patients with prophylactic mastectomy, 13 with prophylactic hysterectomy with salpingoophrectomy, 19 with prophylactic mastectomy with salpingoophrectomy, three with prophylactic colectomy, and three with prophylactic thyroidectomy.

Figures 3 and 4 illustrate the various hereditary cancer syndromes identified through the program. In this cohort, hereditary breast and ovarian cancer syndrome was the most prevalent cancer syndrome identified, accounting for ~62% of all identified genetic conditions, with *BRCA1* PVs being the most common involved gene, followed by Lynch syndrome (with *PMS2* being the most frequently identified genetic cause), followed by familial adenomatous polyposis syndrome and patients with the APC p.I1307K risk allele. Less common syndromes and clinically relevant findings identified include multiple endocrine neoplasia type 2, carriers for *ATM* PVs, Von Hippel‐Lindau syndrome, CHEK2‐associated syndrome, constitutional mismatch repair deficiency, MUTYH‐associated syndrome, PALB2‐associated syndrome, hereditary leiomyomatosis, and renal cell cancer, Cowden syndrome, BRIP1‐associated syndrome, Li‐Fraumeni syndrome, NBN‐associated syndrome, and Neurofibromatosis type 1.

We suggest that the prevalence of Lynch syndrome as well as other less common syndromes such as Familial adenomatous polyposis syndrome, Cowden syndrome, and other cancer syndromes might be underestimated in the cohort, as the program started testing for these syndromes in 2016, while genetic testing related to hereditary breast and ovarian cancer syndrome was started in 2013 and was available locally since 2015.

## 10 RESEARCH AND EDUCATION

### 10.1 Education

The cancer genetic program is heavily involved in education and the program's members, including the medical oncologist and the genetic counselor, had participated and presented in several local and international conferences. Local conferences include precision medicine conferences, breast, gynecology, and colorectal cancer conferences, and the collaborative Doha‐Heidelberg conferences and Qatar Genome symposiums. International conferences in which our program participated include American College of Medical Genetics (ACMG), Canada BRCA symposium: International symposium on hereditary breast and ovarian cancer syndrome, European society of medical oncology (ESMO), and Asia Pacific conference on human genetics (APCHG), in addition to international courses and workshops such as Cancer Genetics courses supported by European Society of Human Genetics (ESHG).

The program also supports local education, such as genomics summer internships and workshops for healthcare providers on hereditary cancer. Moreover, the cancer genetic program heavily supports the main genetic counseling program at Qatar University, which was recently launched in September 2018. Members of the cancer genetic program both provide lectures as well supervision of genetic counseling students during clinical practicums. The cancer genetics program also supports the ACGMEI‐accredited oncology fellowship ACGMEI‐accredited program at HMC.

### 10.2 Research

The cancer genetics program is heavily involved in research within HMC and works collaboratively with other institutions in Qatar including Qatar's University, Sidra Medical Centre, Weill Cornell Medical College, and also helps support Qatar's Genome National Project.

Qatar has a relatively small population, which provides a unique opportunity to strategically coordinate cancer research in a focused and effective manner. Some of the research publications that were produced by the cancer genetics program include studies on prevalence and genotype–phenotype correlations in hereditary breast and ovarian cancer syndromes (Bujassoum, Bugrein, & Al‐Sulaiman, 2017; Bujassoum, Bugrein, Al‐Sulaiman, & Ghazouani, 2016) and Lynch syndrome (Bujassoum et al., 2018). Ongoing research studies include topics such as the impacts of consanguinity on the prevalence of hereditary cancer syndromes in Qatar, the prevalence and genotype–phenotype correlations of Lynch syndrome and other gastrointestinal cancer syndromes, and rare cancer syndromes in Qatar.

## 11 CONCLUSION

The cancer genetics program is an example of a well‐established, multidisciplinary service targeted toward achieving excellence in cancer care through offering prevention, and personalized medicine. This program aligns with Qatar's 2022 vision of achieving excellence in cancer care. With the advances in the field of genomics and precision medicine, programs like the cancer genetics is becoming a core part of the care of patients and their families in Qatar.

The hereditary cancer program is the first of its kind in the Middle East and helps establish HMC as a leader and innovator in patient‐ and family‐centered care, acting as a model for other countries in the Middle East to follow to enhance patient care in the region.

## CONFLICT OF INTEREST

The authors declare that there are no other conflicts of interest.

## DISCLOSURES

S‐BA, R‐AL, H‐B, N‐BA, S‐AK, N‐A are part of the described Section of Medical Oncology, cancer genetics program of National Center of Cancer Care and Research at Hamad Medical Corporation. T B‐O is part of the described section of Clinical and Metabolic Genetics department of Hamad Medical Corporation. F AZ is part of the described section of diagnostic molecular laboratory at Hamad Medical Corporation. H‐G whose part of the Medical Oncology department was responsible of the statistical analysis illustrated in this paper. BDS is an employee of GeneDx, an Opko Health Company, and has stock options in Opko. GeneDx and HMC and the authors work together in multiple clinical and research areas. BDS is the Deputy Editor‐in‐Chief of American Journal of Medical Genetics, part A.
